# NK-92 cells retain vitality and functionality when grown in standard cell culture conditions

**DOI:** 10.1371/journal.pone.0264897

**Published:** 2022-03-16

**Authors:** Rebecca Kotzur, Alexandra Duev-Cohen, Inbal Kol, Adi Reches, Ofer Mandelboim, Natan Stein

**Affiliations:** The Lautenberg Center for General and Tumor Immunology, Institute for Medical Research Israel-Canada, The Hebrew University Hadassah Medical School, Jerusalem, Israel; University of Wisconsin-Madison, UNITED STATES

## Abstract

NK-92 cells are an off-the-shelf, cell-based immunotherapy currently in clinical trials for a variety of cancer types. As the most ‘NK-like’ cell line available, it is also an important research tool. To date, NK-92 cells have been cultivated in a costly and time-consumingly prepared specialized medium, complicating research with these cells. Here we show that NK-92 cells grow in the comparatively user-friendly RPMI medium supplemented with IL-2. We demonstrate that their metabolic activity and replication rates are even improved in RPMI. Furthermore, they can be grown in cell culture dishes and do not need to be expanded in ventilated flasks. We show that in RPMI the cells retain functional characteristics relating to receptor expression, IFN-γ secretion, and killing. Our findings will enable more researchers to work with and manipulate this cell line, hopefully leading to further discoveries and improved therapies.

## Introduction

Natural killer (NK) cells are part of the innate immune system and can be found throughout the body in the blood and in various organs [1]. NK cells are involved in the killing of tumor and pathogenic cells without the need for prior exposure to antigen [2]. Recent findings have described adaptive-like behavior in innate NK cells, hinting at NK “memory” following infection [3, 4].

The human NK cell line NK-92 is highly cytotoxic and IL-2-dependent. It was isolated from a 50-year-old male patient with rapidly progressing non-Hodgkin’s lymphoma [5]. During further characterization, it was noticed that this cell line displays characteristics of activated NK cells in vivo, including the expression of CD56, activating cell surface receptors, and the NK cell typical adhesion molecule CD2 [5]. Given their strong resemblance to NK cells, these cells are currently in clinical trials as an off-the-shelf cellular immunotherapy for a variety of cancers. This is seen as a novel approach in immunotherapy due to their prolonged activation after irradiation, nontoxicity towards healthy cells, low immunogenicity, and ability to be easily and rapidly expanded in culture. These cells have proven highly effective against both solid and liquid cancers, as well as virally infected cells due to their functionality even after irradiation and IL-2 withdrawal for 72 h [6, 7]. NK-92’s ability to be produced from a master cell bank and expanded within days for clinical use makes them a highly valuable form of cellular immunotherapy. So far, they have already been used in clinical trials and their safety has been demonstrated in various application [8].

NK-92 cells were found to express virtually all major NK activating receptors, lacking only CD16 surface expression. Additionally, they express only the killer cell immunoglobulin-like receptor (KIR) family member KIR2DL4 and none of the other KIR-family members, which are characterized as inhibitory receptors. Activating receptors like NKp30 were found to be strongly expressed on the surface, and NKp44 is expressed at lower levels. Additionally, CD2 and CD56, both NK cell adhesion molecules, have been found to be expressed on the surface of NK-92 cells. This is just a partial list of activating receptors displayed on the surface of NK-92 cells [5]. NK-92 cells have demonstrated the ability to lyse tumor cells in vitro, further expanding their potential application [5, 9, 10]. Given their wide repertoire of receptors, coupled with their Interferon (IFN)γ-secreting and cytotoxic abilities, NK-92 are viewed as the most “NK cell-like” cell line in the clinical and research usage. It also makes them a desirable model for research due to their ability to be transformed and cultured far more efficiently than primary NK cells.

Several types of transformed NK-92 cells are currently in clinical research and use and can be divided into different groups: NK-92MI cells, haNKs (high affinity), taNKs (targeted affinity) and t-haNKs, a hybrid of the transfectants. NK-92 are dependent on exogenous IL-2, so NK-92MI were engineered to produce autologous IL-2 [11].

haNKs are engineered to express CD16, the only major activating receptor which native NK-92 cells lack. This allows for an increase in their ADCC activity as well as aiding proper function within the patient [12].

taNKs are modified to specifically target ligand expression on tumor cells, analogous to chimeric antigen receptor (CAR) T cells. They are essentially off-the-shelf CAR NK-cells, cultured with the aim of recognizing and killing only tumor cells. A cell line targeted against ErbB2-postive breast carcinoma has already shown great potential in treating this disease [13]. In contrast to primary human NK cell derived CAR-NK cells, taNKs cells can be easily expanded in regular cell culture, making this therapy more widely applicable. The establishment of CAR-NK generation with NK-92 cells, would push this form of cancer treatment towards a new wave of applications [14]. Several studies have already described CAR-modified NK-92 cells and their efficacy [15–19]. t-haNKs are engineered to combine the features of all transfectants in one. The cells express high-affinity CD16, secrete IL-2, and are modified to recognize specific tumor cell ligands, for example PD-L1 [20].

All these transfectants illustrate the potential of investigation of NK-92 cells, since they are easier to work with than primary cells, more expandable in culture, and preferrable for transfection to create desired effects.

One main disadvantage of NK-92 cells is the relatively complex and expensive cell culture medium essential for cell growth. The standard medium requires several special ingredients, use of a chemical hoods, filtering, and protection from light, among other drawbacks. Here we describe the continuity of NK-92 cells’ positive properties even in a simple, standard growth medium—RPMI.

We demonstrate that growing NK-92 cells can be easier, less time consuming, and more cost effective, all while maintaining their cytotoxic and secretory potential. This will support the clinical and research utility of these cells by making them even more convenient for use.

## Material and methods

### Cell lines and growth conditions

K562, RAJI, 8866, C1R and 721.221 cells were maintained in RPMI (Sigma-Aldrich) supplemented with 10% fetal calf serum (Sigma-Aldrich), 2 mM glutamine (Biological Industries (BI)), 1 mM sodium pyruvate (BI), 1× nonessential amino acids (BI), 100 U/ml penicillin (BI), 0.1 mg/ml streptomycin (BI).

RKO, T-47D, and A549 cells were maintained in DMEM (Sigma-Aldrich) supplemented with 10% fetal calf serum (Sigma-Aldrich), 2 mM glutamine (Biological Industries (BI)), 1 mM sodium pyruvate (BI), 1× nonessential amino acids (BI), 100 U/ml penicillin (BI), 0.1 mg/ml streptomycin (BI).

NK-92 cells were maintained in two different media: RPMI (Sigma-Aldrich) supplemented with 10% fetal calf serum (Sigma-Aldrich), 2 mM glutamine (Biological Industries (BI)), 1 mM sodium pyruvate (BI), 1× nonessential amino acids (BI), 100 U/ml penicillin (BI), 0.1 mg/ml streptomycin (BI), and 200U/ml IL-2 (PeproTech); or MEM-alpha (BI) supplemented with 12.5% fetal calf serum (Sigma-Aldrich), 12.5% Horse serum (BI), 2 mM glutamine (BI), 1 mM sodium pyruvate (BI), 1× nonessential amino acids (BI), 100 U/ml penicillin (BI), 0.1 mg/ml streptomycin (BI), 200 U/ml IL-2 (PeproTech), 0.2 mM myoinositol, 0.02 mM folic acid, 0.1mM beta-mercaptoethanol, 0.2% Ribonucleosides and Deoxyribonucleosides for MEM-Alpha.

### Activation assays with IFN- *γ* and TNF-*α*

To assess the activation capacity of the NK-92 cells in different conditions, NK-92 cells were incubated for 48h in their respective medium at a 1:1 ratio with 50,000 target cells—721.221, A549, C1R, K562, RAJI, RKO, and T47D at 37°C. Additionally, the interleukins IL-12, IL-15, IL-18 and combinations of those (IL-12 + IL-18, IL-12 + IL-15 + IL-18) as well as LPS were used for activation at the following concentrations—IL-12 20ng/ml, IL-15 500ng/ml, IL-18 100ng/ml and LPS 5μg/ml. The cell-free supernatant was used for IFN-γ and tumor necrosis factor (TNF)-*α* specific sandwich ELISA. Nunc MaxiSorp^™^ flat-bottom ELISA plates (Invitrogen) were coated with 1 μg/ml purified anti-IFN-γ (BLG-502402) or anti- TNF-*α* (BLG-502802) in 50 μl PBSx1 and incubated for 2h at 37°C followed by blocking with 200 μl 1% BSA in PBSx1 incubated for 2h at room temperature (RT). Washing buffer of PBSx1 + 0.05% Tween-20, was used for washing the wells 3 times. 100 μl of supernatant were incubated within the coated wells at 4°C overnight. The biotinylated IFN-γ detection antibody (BLG-502504) or TNF-*α* detection antibody (BLG-502904) was then added at 1 μg/ml in 100 μl 1% BSA in PBSx1 and incubated for 1h at RT. Finally, streptavidin HRP (016-030-084, Jackson immuno research) 1μg/ml in 100 μl PBSx1 + 0.05% Tween-20 + 1% BSA was incubated for 30 min at RT, and quantification was performed with TMB one component substrate (Southern Biotech).

### Killing assays

For assessment of functional killing of NK-92 cells compared between the different media and growth conditions radioactive killing assays were employed. For the assay K562, RAJI, 8866, and 721.221 cells were labelled with [^35^S]-Methionine 12h prior to the assay as target cells. The labelled targets, 5,000 cells/well, were incubated with the NK-92 cells in different conditions and various E:T ratios (2:1, 8:1) as effector cells. The assays were performed in RPMI + IL-2 or NK-92 medium in 96-U shaped plates at 37°C overnight. After incubation, the plates were centrifuged (1600rpm, 5min, 4°C) and 50μl of the supernatants were collected and transferred to opaque Opti-plates (Packard). Following the addition of 150μl scintillation liquid (Perkin Elmer) the plates were analyzed by a micro beta, β-counter (Perkin Elmer). The maximal [^35^S] release was determined by adding 100 μl of 0.1N NaOH to each target cell line. Spontaneous release of radioactivity was determined in wells containing target cells only. The final specific lysis was calculated as follows: ((radioactive reading − spontaneous release)/(maximal release − spontaneous release))*100 = specific lysis.

### Staining of receptors

In order to verify the surface expression of various receptors on NK-92 cells FACS stainings were conducted. The cells were incubated on ice for 30 min with 0.5 μg of conjugated antibodies per 1x10^5^ cells in 100 μl FACS medium. For purified unconjugated antibodies 0.5 μg were used for 60 min on ice followed by a secondary antibody at a 1:200 dilution for 30 min. Antibodies used are listed in Table 1. For each antibody corresponding isotype controls were used. After two washing steps the stained cells were strained through a mesh and the fluorescence measured by the Cytoflex Flow Cytometer and analyzed by FACSexpress Version 6.

**Table 1:** Antibodies.

2B4 APC	Biolegend	BLG-329512
CD158 PE	Life sciences	LS-C16157-100
CD16 APC	Biolegend	BLG-360706
CD2 purified	biorad	MCA1194
CD56 APC	Biolegend	BLG-318310
CD84 PE	Biolegend	BLG-326008
CEACAM-1 PE	Biolegend	BLG-342304
DNAM-1 APC	Biolegend	BLG-338312
KIR2DL4 PE	RnD	FAB2238P-100
NKG2D PE	Biolegend	BLG-320806
NKp30 APC	Biolegend	BLG-325210
NKp44 APC	Biolegend	BLG-325110
NKp46 APC	Biolegend	BLG-331918
NTB-A PE	Biolegend	BLG-317208
PD-1 PE	Life span	LS-C125334
TIGIT APC	Biolegend	BLG-372706
Goat anti Mouse 647	Jackson immuno research	115-606-062
NKG2A APC	RnD	FAB1059A-025
NKG2C APC	RnD	FAB138A-100

### Viability assays

#### MTT assay

In order to evaluate the proliferation of the NK-92 cells in different conditions M5655 Sigma thiazolyl blue tetrazolium bromide (MTT) was used. 50 mg MTT was solubilized in 10 ml PBSx1 and sterile filtered. For each condition 25,000 cells per well were seeded at day 0 in quadruplicates in the respective media. After 24h intervals the cells were incubated with 10 μl of MTT for 3h in 37°C up until 72h after initial seeding. Following the incubation, cells were resuspended in 100 μl DMSO to lyse the stained cells. After cell lysis, the colorimetric changes were measured at 590 nm.

#### CFSE cell proliferation assay

1 × 10^6^ cells/mL NK-92 cells from all growth conditions were labeled with CFSE at a final working concentration of 10 μM, according to manufacturer’s instructions (CellTrace^™^ CFSE Cell Proliferation Kit, Thermo Fisher), and incubated for 10 min at 37°C. The staining was quenched by using 5 volumes of ice-cold culture medium. After incubation for 5 min on ice and washing of the cells, the cells were distributed to 2.5 x 10^4^ cells per well according to their growth conditions. The stained cells were measured in the cytoflex at 24h intervals until 48h after initial labeling and analyzed with FACSexpress Version 6.

### Statistical analysis

Two-tailed ANOVA was performed with GraphPad PRISM 6 software. Multiple unpaired Student’s T test was two-tailed with the assumption of heteroscedasticity.

## Results

### NK-92 cells can be grown in RPMI with IL-2

**Fig 1:**
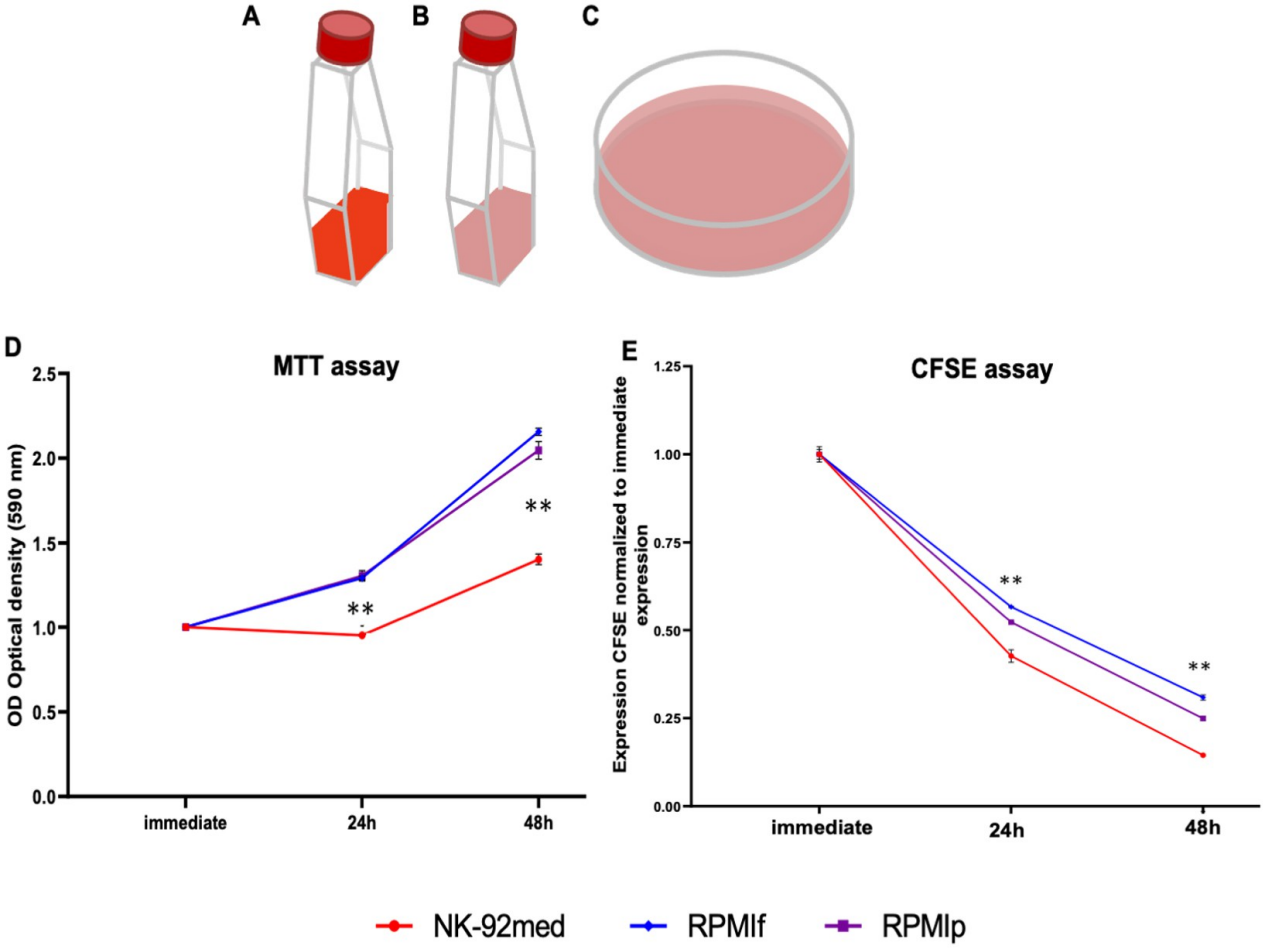
Schematic representation of the growth conditions and media of the NK-92 and growth progression in the media. A) Standing T25 flask containing the specialized NK-92 medium, which is in standard use (**NK-92med**, medium represented in red); B) Standing T25 flask containing RPMI medium with added IL-2 **(RPMIf, RPMI is represented in pink**); C) 10cm tissue culture dish containing the general RPMI-IL-2 medium (**RPMIp, RPMI is represented in pink**). D) MTT assay is shown over the course of 48h of incubation. Measurements are of optical density (OD) of a sample drawn at the indicated time after changing of medium. E) CFSE assay, as measured by FACS. Hours indicate time after addition of CFSE. Shown is the staining with CFSE normalized to the staining immediately upon addition of CFSE to the medium. D and E show 1 representative out of 3 assays performed. red = **NK-92med**., blue = **RPMIf**, violet = **RPMIp** *p<0.05, **p<0.005 compared between the NK-92 in NK-92 medium as control and the NK-92 in RPMI + IL-2 medium (two-tailed ANOVA test).

We set out to determine if NK-92 cells are viable and retain their basic properties when cultured in more convenient growth medium and plasticware. Over the course of this study, we kept NK-92 cells in three growth conditions. The first was our “standard care” group, used as a control. These cells were grown in conditions consistent with usual practice—small, standing, ventilated T25 cell culture flasks with the specialized NK-92 medium containing IL-2. We will refer to these cells as “**NK-92med**” in reference to their specialized medium. The second group was NK-92 in ventilated T25 cell culture flasks, but with RPMI medium along with IL-2. These will be denoted **RPMIf** (“f” for flask). Finally, we grew NK-92 cells in the RPMI-IL-2 medium, but in 10 cm cell culture petri dishes, denoted **RPMIp** (“p” for plate) (illustrated in Fig 1A–1C). All cells were incubated at 37°C and 5% CO_2_. The cells were grown in 5 ml of medium in the T25 flasks and in 10–12 ml of medium in the petri dishes. All experiments were carried out on NK-92 cells which had been cultured in the stated conditions for at least six months.

We also compared the cost differences between the media, listed in Table 2. We compared the price per 500 ml, the number of ingredients used to produce the final medium in standard research facilities, the necessity of light protection for the final product, safety regards while preparing the media, and the requirement for sterile filtering. Our comparison shows that the specialized NK-92 medium is significantly more expensive and requires almost twice as many ingredients as does the RPMI medium. Additionally, as the specialized medium contains beta-mercaptoethanol its preparation requires the use of personal protection, namely a chemical hood. Both the beta-mercaptoethanol and the folic acid contained in the medium require it to be protected from light. Despite all of these differences, the specialized NK-92 medium does not appear to allow for a higher cell density when culturing the NK-92 cells when observed in culture.

**Table 2:** Features of the different media used in this study.

	NK-92 medium	RPMI+IL-2
**Price/500 ml**	43 USD	9 USD
**Number of ingredients**	12	7
**Protection from light**	Yes	No
**Chemical hood required**	Yes, contains beta-mercaptoethanol	No
**Requirement for sterile filtering**	Yes	No

NK-92 medium, widely used research with NK-92, and RPMI medium with added IL-2, a novel approach to make research with NK-92 cells more feasible. Compared are the price per 500 ml of each medium, the number of ingredients, necessity of light protection, safety regards concerning the preparation of the medium, and the requirement for sterile filtering before use.

### Cell viability is preserved in alternative growth conditions

Next, we assessed the viability of NK-92 cells in the different media. We did this using two different experimental approaches. First, we performed an MTT assay (Fig 1D), which assesses the metabolic activity of the investigated population. Enzymes within the cells reduce MTT, leading to a change in color, which we quantified over a time course of 48h. For more details, please see the Materials and Methods section. The MTT assay shows an increase in cell number at 24h for cells grown in RPMI, with a doubling by 48h (Fig 1D). The cells in NK-92 medium showed significantly reduced metabolic activity compared to those in RPMI.

We then utilized a CFSE assay (Fig 1E). Here we saw continuous proliferation of cells and decrease of CFSE until 48h in all conditions (Fig 1E), with a statistically significant increase of growth in the NK-92 cells grown in NK-92 medium compared to the RPMI medium. It is worth noting that in our hands we did not perceive such a difference in proliferation in culture. Nevertheless, this difference did not influence our work. Both assays showed maintenance of proliferation and metabolic activity in cells cultured in RPMI. The MTT assay even suggested higher metabolic activity in the NK-92 cells cultivated in RPMI, but the CFSE assay shows a slightly higher proliferation activity in the NK-92med cells.

### NK-92 retain their characteristic receptor surface expression, regardless of growth condition used

NK-92 cells are characterized by a set of surface proteins utilized as markers. Gong et al. characterized NK-92 via specific surface markers and their expression levels, for example positive for CD56^bright^ and CD16 negative, and checked for various immune cell specific cell markers, including both activating and inhibitory NK cell receptors [5].

**Fig 2:**
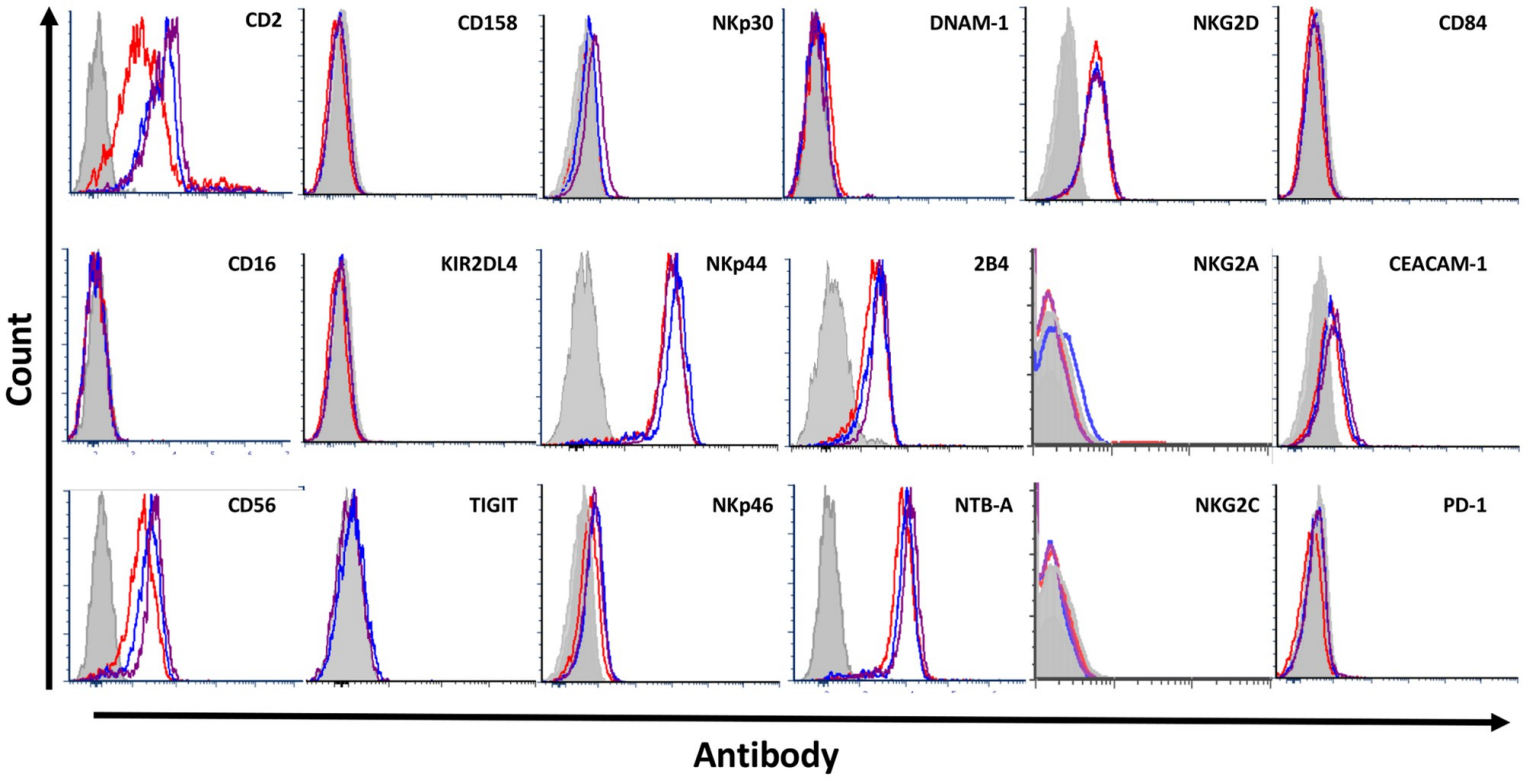
Characterization of surface receptor expression. 1 out of 4 representative FACS stainings against various inhibitory and activating NK cell markers (CD2, CD56), KIR (CD158, KIR2DL4), activating receptors (NKp30, NKp44, NKp46, NKG2D, NKG2C, DNAM-1, 2B4, NTB-A, CD84), and inhibitory receptors (TIGIT, CEACAM-1, PD-1, NKG2A) grey = background staining, red = **NK-92med**., blue = **RPMIf**, violet = **RPMIp**.

To assess the surface characteristics of the NK-92 cells grown in different conditions, we first conducted a FACS staining for selected surface markers of NK-92 along with the main activating and inhibitory receptors expressed by NK cells. We compared expression of these markers between growth conditions (Fig 2). Our stainings showed no major differences in the expression of surface receptors across growth conditions. We observed a slight elevation of the expression of the NK markers CD2 and CD56 in cells cultured in RPMI, but no significant changes in the functional receptors.

### Activation potential of NK-92 cells is preserved in alternative growth conditions

We next looked to assess the functional capacity of the cells in the different growth conditions.

**Fig 3:**
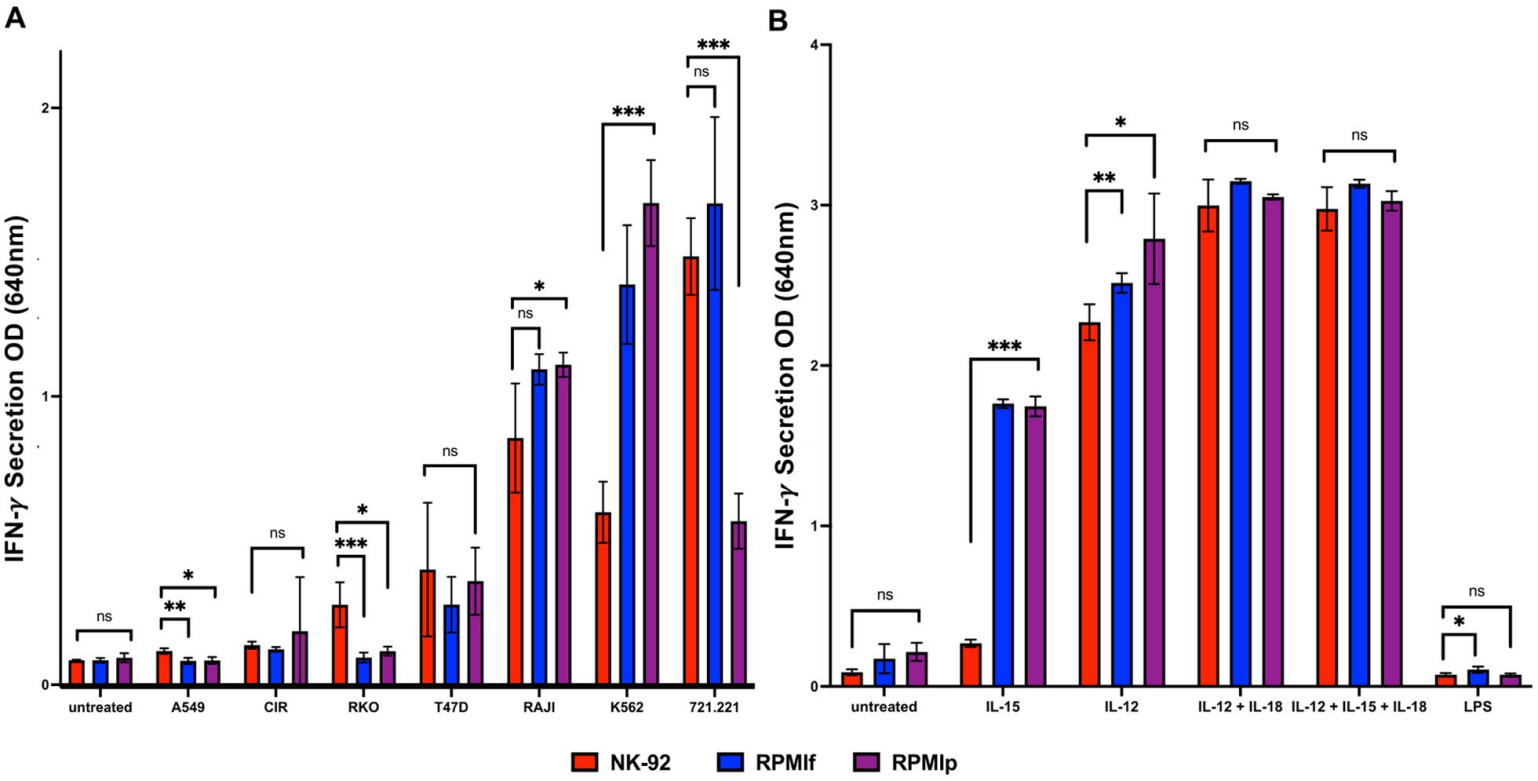
Comparison of secretory potential after activation of NK-92 cells in different growth conditions. A) + B) Secretion of IFN- γ; A) Secretion after activation with tumor cells for 48h at 37°C (721.221, A549, C1R, K562, RAJI, RKO, T47D), B) Secretion after activation with interleukins after 48h of incubation at 37°C (IL-12, IL-15, IL-12 + IL-18, IL-12 + IL-15 + IL-18, LPS); red = **NK-92med**., blue = **RPMIf**, violet = **RPMIp**. *p<0.05, **p<0.005, ***p<0.001 compared between the NK-92 in NK-92 medium as control and the NK-92 in RPMI + IL-2 medium (two-tailed ANOVA test + multiple unpaired T-tests). Shown is 1 representative out of 5 assays performed.

In order to do so, we incubated the cells with different cell lines as targets. As NK-92 cells express a wide variety of NK activating receptors, they can recognize many different types of cancer cells. Cells were co-incubated and then the supernatant was used for a sandwich-ELISA directed against IFN-γ. The cell lines used were: 721.221, A549, C1R, K562, RAJI, RKO, and T47D (Fig 3). Depicted in Fig 3A is the secretion of IFN-γ, which showed mostly non-significant differences. Only incubation with K562 and RAJI showed significant differences, in both cases favoring the cells in RPMI. With 721.221 cells, we saw an unexplained reduction in secretion by the RPMIp cells, not seen in RPMIf.

Additionally, we used a variety of secreted factors which are known to stimulate NK cells. We used the interleukins IL-12, IL-15, and IL-18 alone or in combination as well as LPS from bacterial membrane molecules (Fig 3B). As depicted in Fig 3B, some significant differences were observed with the incubation and activation by interleukins, notably of IL-15. All the interleukins and interleukin combinations showed increased secretion of IFN-γ in the cells incubated with the RPMI + IL-2 medium. In contrast, the incubation with LPS did not show any activation of IFN-γ secretion.

Additionally, we employed TNF-*α* as a second cytokine to assess the activation of the NK-92 cells. Depicted in S1 Fig, the same supernatants used for the IFN-γ assay did not show any appreciable increases in TNF-*α* concentration with stimulation. Therefore, the determination of activation via TNF-*α* was abandoned and focused on IFN- γ.

### Cytotoxic potential is preserved across growth conditions

**Fig 4:**
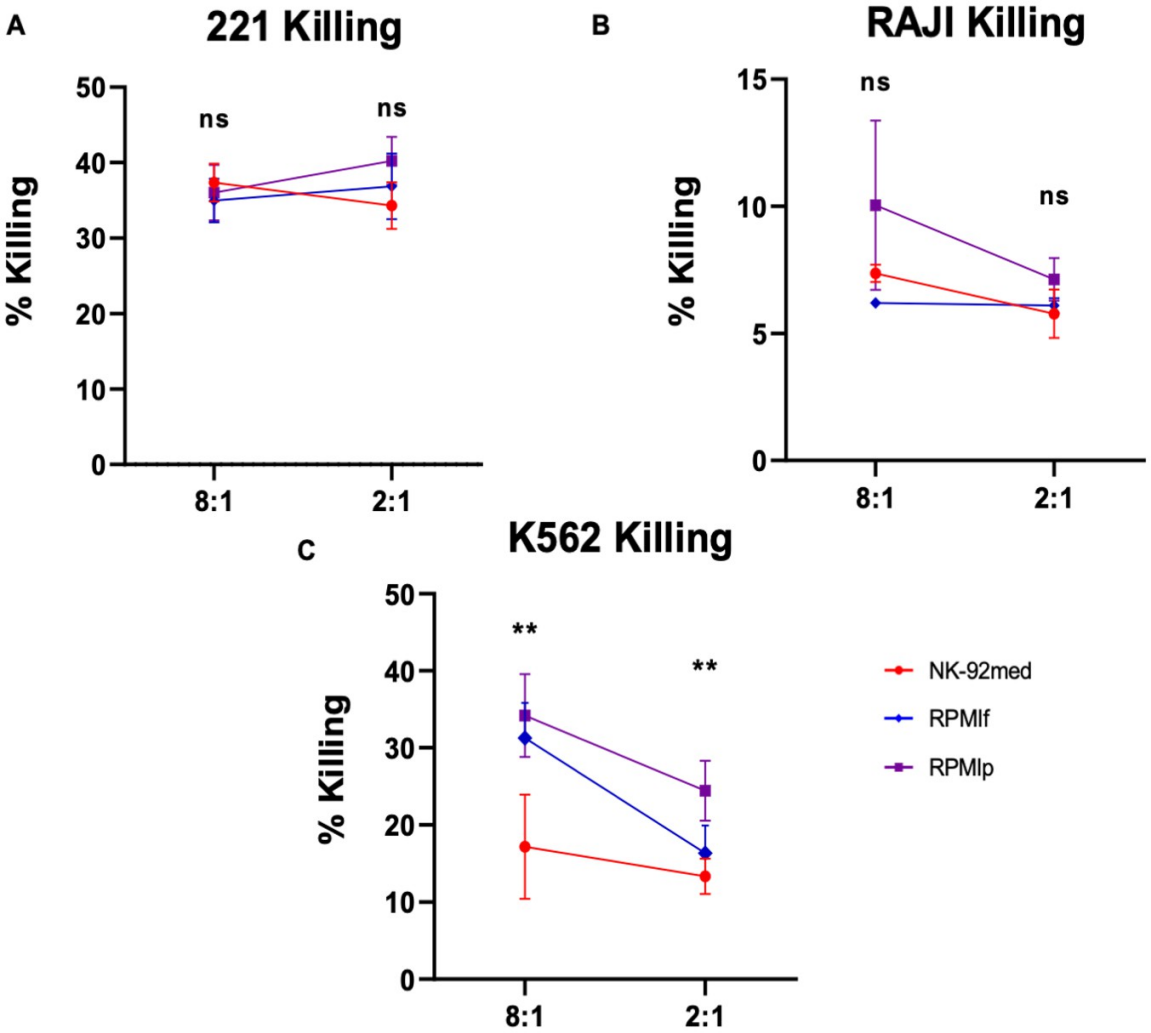
Comparison of killing potential. Killing of A) 721.221 cells, B) Raji cells, and C) K562 cells. E:Ts: 8:1, 2:1 red = **NK-92med**., blue = **RPMIf**, violet = **RPMIp**. *p<0.05, **p<0.005 compared between the NK-92 in NK-92 medium as control and the NK-92 in RPMI + IL-2 medium (two-tailed ANOVA test). Shown is 1 representative out of 5 killing assays performed.

Due to the nature of the NK-92 cells and their therapeutic usage, we also elucidated their cytotoxic potential in the different growth conditions. In order to assess their functionality in vitro, we employed a killing assay displaying the killing efficiency by increase of radioactive signal. We used [^35^S]-methionine to label target tumor cells and incubated them with the NK-92 cells as effector cells at both low (2:1) and high (8:1) E:T ratios. Release of radioactively labelled proteins to the supernatant was used as a measurement of target cell-killing. RAJI, K562, and 721.221 cells were used as targets. In Fig 4A, we see no loss of killing potential with 721.221 cells when NK-92 cells are grown in RPMI. As these 721.221 cells are strongly killed by NK cells, the killing appears saturated even at low E:T ratios. In Fig 4B, with RAJI cells, we found similarly elevated killing in RPMI as compared to the NK-92 cells grown in NK-92 medium, though this difference was not significant. When using K562 cells as targets, we observed significant differences between the standard condition and the RPMl conditions, with the cells grown in RPMI-IL-2 medium showing an increase in killing (Fig 4C).

Raw data for all plots can be found in S1 Table.

## Discussion

As expected, NK-92 cells grown in our hands in the specialized NK-92 medium in the recommended standing T25 flasks propagated well and maintained the characteristics described by the Klingemann lab in 1994 [5], even after long periods of continuous culturing. Here we show that the same cells grow as well or even better in the widely available RPMI with added IL-2, both in the recommended T25 flasks and the commonly used 10cm tissue culture dishes. The cells we used were not modified in order to grow better in the RMPI medium but were standard NK-92 cells. Cell viability assays showed no disadvantage of growth in the alternative novel conditions. Surface staining revealed no significant differences in the expression of the essential surface receptors of NK-92 cells compared between the different growth conditions. Activation assays with various tumor cell lines and interleukins show non-inferior and at times even superior IFN-γ secretion by cells cultivated in RPMI. Finally, functional killing assays showed equivalent or increased killing capacity of tumor cell lines when cells were grown in RPMI.

Parental NK-92 cells are currently in stage II clinical trials for a variety of malignancies. Some of the transfectants described above are also being investigated clinically, and several others are in pre-clinical development. This pool of potential therapies highlights the versatility of this drug. As a cell line, many labs can modify it to target a specific disease (different iterations of CAR-NK-92 cells) or improve NK-92 function (i.e. NK-92MI). While this has always been a possibility, the cumbersome nature of work with these cells has no doubt held the field back.

New therapeutic strategies are constantly being developed in hundreds of laboratories across the world. As evidenced by the very limited availability and astronomical costs of CAR-T cell therapy, the jump from bench to bedside is quite a long one. Using an off-the-shelf therapeutic like NK-92 can help patients quickly get the solutions they desperately need. Making this tool accessible by using reagents already found in virtually all of these labs will help facilitate the process of swiftly translating scientific findings into clinical solutions.

During our research we became aware of several labs investigating the effects of media on primary NK cells along with their influence on the purification process of those cells for immunotherapy [21, 22]. This, too, demonstrates the need for cost-efficient growth conditions for the cells destined for immunotherapy, as research on off-the-shelf cell therapeutics will only continue to increase worldwide. These findings also suggest NK-92, formerly thought to be challenging to grow, can be cultured under various conditions. In previous publications on the topic, however, the focus was rather on optimized purification methods, but less on the proof of equal functionality of NK-92 in different media [22]. We present comprehensive evidence that interchangeable growth medium does not inhibit, and in some cases even slightly improves, NK-92 activity.

The innovation surrounding the NK-92 cell line is virtually endless with new approaches in every direction due to the possible manipulation of these cells in these quantities. For example, it is possible to increase the activity of NK cells by modifying haNKs to work in hypoxic environments such as those commonly found in the tumor microenvironment which usually hamper the endogenous NK cell activity [23]. Also, the usage of PD-L1 CAR NK cells is made possible with this cell line, which is ready to be translated to clinical studies showing cytotoxic behavior against human head and neck squamous cell carcinoma in mice, demonstrating crosstalk with the adaptive immunity [24]. These are only a few examples of recent scientific findings enabled by the use of NK-92 cells.

NK-92 cells are a vital tool in immunotherapy. They enable labs around the world to utilize their specific knowledge to manipulate this cell line and create innovative ways to cure malignancies that were thought to be untreatable. They pave the way to affordable research in the field of clinical therapy, since the generation of mutants and transfectants made to influence the cells’ functionality is made possible in the most NK-cell like line we have at our disposal. We further investigated this tool to make research even easier and innovations more attainable. The versatility and easier handling of NK-92 allows labs to implement all of their specifically investigated aspects of NK cell reactivity towards hostile invading cells. These specific modifications allow for directed targeting of cancer markers, which are discovered in an increasing rate by researchers around the globe.

Clinical trials utilizing the modified NK-92 cell lines already include good manufacturing processes (GMP) which entails proper and continuous documentation of methods and materials used, ensuring of consistent material and product quality and the use of GMP-compliant materials. Compliance with these guidelines ensures the quality and safety of the product, not only in the finished version, but throughout the entire production process. This is employed for all trials using NK-92 cells for usage in human participants. One example of GMP-compliant maintenance of NK-92 before injection into human patients is described in Williams et al. by using GMP-certified ingredients including X-Vivo 10 medium, human serum, human recombinant IL-2, and L-asparagine, L-glutamine and L-serine [19]. Very strikingly, the usage of antibiotics or immediate harmful substances does not comply to GMP-standards. Before irradiation, the cells are resuspended in a GMP-compliant isotonic injection solution supplemented with human serum and then, after irradiation, injected into the patient [19]. Other groups use similar products, namely: (1) serum-free medium, (2) human serum, and (3) human recombinant IL-2 [25]. Along with the materials, the facilities preparing the NK-92 cells must comply GMP standards in order to ensure optimal adherence to the regulations. Additionally, the quality of the product is assessed before application into the patient. 0.5–1.5x10^8^ cells are infused into the patient, with the exact dose dependent on weight. In order to achieve these amounts of cells, the cells have to be grown in culture bags [19]. To implement all of the mentioned criteria requires meticulous planning of the usage of NK-92 cell lines in clinical applications and the transference from the bench to the bedside. Research can be done in the established media, but the influence of the medium on the performance of cells cannot be underestimated and must be considered accordingly.

All in all, we have shown that NK-92 cells—known for their research and clinical utility—are more versatile than previously thought. Various clinical trials are underway in the US and Germany to evaluate the efficacy of these cells alone or in combination with established immunotherapy reagents. Our findings will aid in furthering future research on these cells. Our methods are simpler, less time consuming, and more cost effective than conventional methods used to maintain these cells.

## Supporting information

S1 Fig Comparison of secretory potential via TNF*α* after activation of NK-92 cells in different growth conditions: A) + B) Secretion of **TNF*α***; A) Secretion after activation with tumor cells for 48h at 37°C (721.221, A549, C1R, K562, RAJI, RKO, T47D), B) Secretion after activation with interleukins after 48h of incubation at 37°C (IL-12, IL-15, IL-12 + IL-18, IL-12 + IL-15 + IL-18, LPS); red = **NK-92med**., blue = **RPMIf**, violet = **RPMIp**. *p<0.05, **p<0.005, ***p<0.001 compared between the NK-92 in NK-92 medium as control and the NK-92 in RPMI + IL-2 medium (Two-tailed ANOVA test + multiple unpaired T-Tests). Shown is 1 representative out of 3 assays performed.
(TIF)

S1 Table Values used to build graphs: Means, standard deviations and N-value for each presented graph as well as unedited raw data presented. (XLSX)
