# Supplementary figures and images for: NK-92 cells retain vitality and functionality when grown in standard cell culture conditions
Source: PLoS One. 2022 Mar 16;17(3):e0264897. doi: 10.1371/journal.pone.0264897 (PMC8926178; doi:10.1371/journal.pone.0264897)

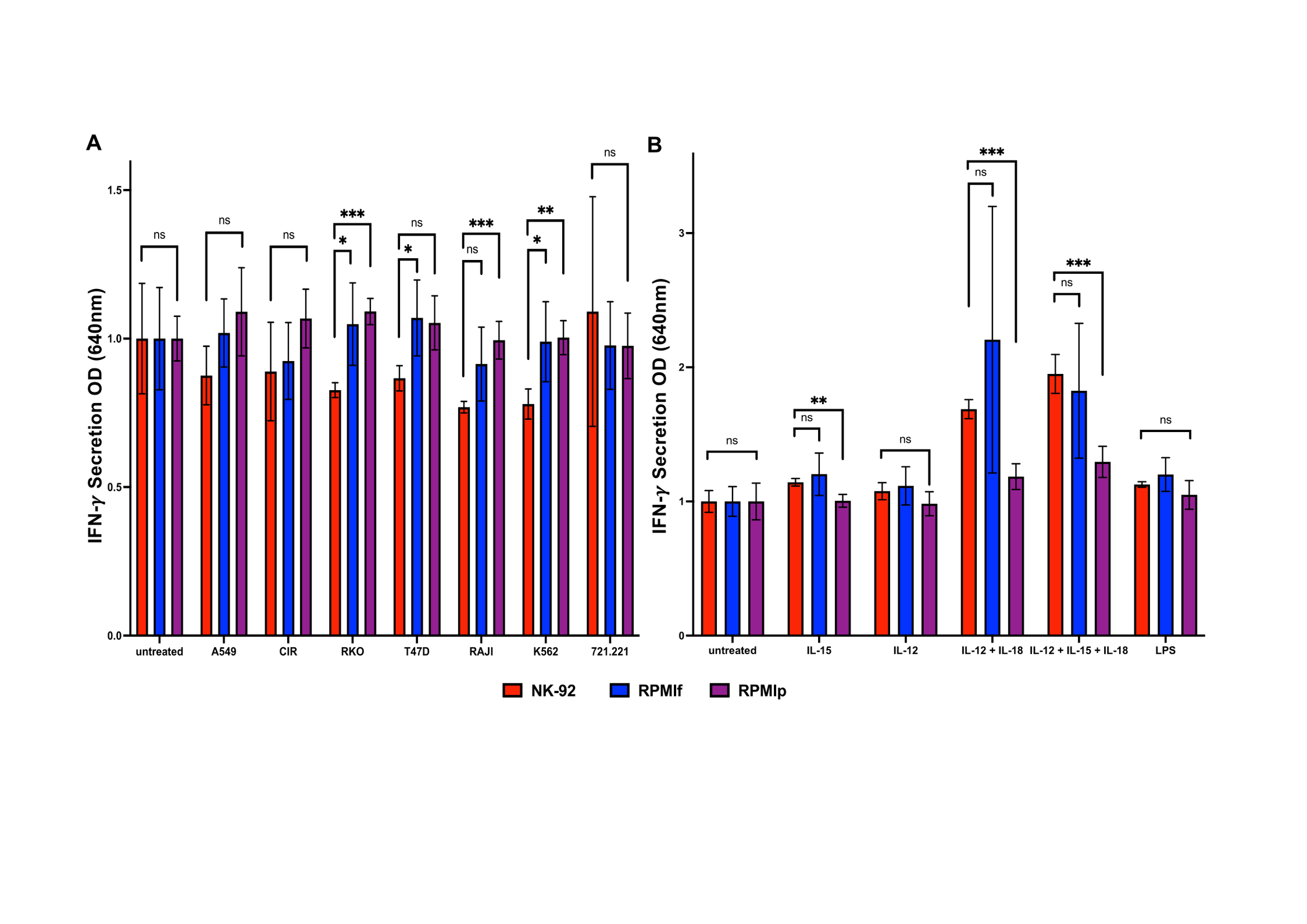

Supplement: S1 Fig — Comparison of secretory potential via TNFα after activation of NK-92 cells in different growth conditions: A) + B) Secretion of TNFα; A) Secretion after activation with tumor cells for 48h at 37°C (721.221, A549, C1R, K562, RAJI, RKO, T47D), B) Secretion after activation with interleukins after 48h of incubation at 37°C (IL-12, IL-15, IL-12 + IL-18, IL-12 + IL-15 + IL-18, LPS); red = NK-92med., blue = RPMIf, violet = RPMIp. *p<0.05, **p<0.005, ***p<0.001 compared between the NK-92 in NK-92 medium as control and the NK-92 in RPMI + IL-2 medium (Two-tailed ANOVA test + multiple unpaired T-Tests). Shown is 1 representative out of 3 assays performed. (TIF) [file pone.0264897.s001.tif]
