# Supplementary material for: NK-92 cells retain vitality and functionality when grown in standard cell culture conditions
Source: PLoS One. 2022 Mar 16;17(3):e0264897. doi: 10.1371/journal.pone.0264897 (PMC8926178; doi:10.1371/journal.pone.0264897)
Supplement: Attachment — Submitted filename: Response to reviewers.docx [file pone.0264897.s003.docx]

Response to reviewers

Dear editors and reviewers,

In the presented manuscript titled **NK-92 cells retain vitality and functionality when grown in standard cell culture conditions** submitted by Kotzur et al., the influence of the selected growth medium used for growing and maintaining the NK-92 cell line on the vitality and functionality of the cells is assessed. During the study, the investigators found no significant disadvantage in both factors while using the standard culture medium RPMI supplemented with IL-2 compared to the recommended specialized NK-92 growth medium for long term culturing. This led to the conclusion that supplemented RPMI medium can be used for everyday culturing of NK-92 cells replacing the comparably more expensive and resource-consuming NK-92 medium.

After first submission, the authors answer to the comments raised by the reviewers and editor as follows:

1. The introduction should be more concise. It looks redundant with descriptions on clinical trials that are not directly relevant to the purpose of the comparison study.
   a. We have shortened the introduction in accordance with the reviewer’s suggestion.
2. To minimize necessary supplement or treatment is important for cost effectiveness, however, data obtained through preclinical studies using GMP-compliant reagents will eventually required for the next step. One may not expect products prepared in petri dishes will be administered to patients even in early phase of study. Careful planning could be also critical.
   a. The reviewer is, of course, correct that GMP compliance is required for clinical administration. One of the main points of this paper, however, is to enable expansion of the use of NK-92. GMP facilities are not accessible to many groups (largely including our own) and are certainly not required in the exploratory research we wish to encourage. We have put forward the concept of easier handling, but ultimately the investigators in a clinical trial should, of course, carefully consider whatever methods they choose to employ.
3. Please ensure that your manuscript meets PLOS ONE's style requirements, including those for file naming.
   a. We have made the necessary changes.
4. Thank you for stating the following in the Acknowledgments Section of your manuscript:
   a. This work was supported by a network grant of the European Commision (H2020-MSC_ITN_765104_MATURE-NK) to the Mandelboim lab, the Israel Innovation Authority (Kamin grant), the Israel Science Foundation (Moked grant), the GIF Foundation, the ICRF professorship grant, the ISF Israel- China grant, the MOST-DKFZ grant, and the Ministry of Science and Technology grant.
   b. Please note that funding information should not appear in the Acknowledgments section or other areas of your manuscript. We will only publish funding information present in the Funding Statement section of the online submission form.
      Please remove any funding-related text from the manuscript and let us know how you would like to update your Funding Statement.
      i. We have made the necessary changes, thank you for pointing this out!
5. It should be clarified whether the potential of NK-92 cells are maintained after long-term cultivation in the RPMI-based media.
   a. All experiments were carried out on NK-92 cells which had been cultured in the stated conditions for at least six months. This is now indicated in the manuscript.
6. The authors should present another form of activation used for the activation assay depicted in Fig. 3. A bacterial molecule like LPS could enhance the significance of the findings.
   a. A variety of cellular targets, interleukins (alone or in combination), and LPS are all featured in Figure 3, as requested by the reviewer.
7. As well, another inflammation associated cytokine apart from IFN should be added as an output marker for activation of NK-92 cells in Fig. 3, for example TNF-a.
   a. Results of TNF$\alpha$ secretion are now included in the manuscript summed up in Supplementary Figure 1.
8. The authors show in Fig. 2 a depiction of selected surface markers known to be expressed on NK-92 cell, but in the manuscript text many more markers are mentioned. Please add more marker staining to complete the picture of unfazed cells after the medium exchange.
a. Please see the manuscript for the full, updated list in Figure 2.

We thank each of the reviewers and the academic editor for their valuable comments and implemented the raised improvements in the current re-submitted manuscript.
