# Supplementary material for: NK-92 cells retain vitality and functionality when grown in standard cell culture conditions
Source: PLoS One. 2022 Mar 16;17(3):e0264897. doi: 10.1371/journal.pone.0264897 (PMC8926178; doi:10.1371/journal.pone.0264897)
Supplement: Peer review history

# Peer review: Decision Letter 0

**DOI:** 10.1371/journal.pone.0264897.r001

**Authors:** Yoshiki Akatsuka

19 Oct 2021

PONE-D-21-23659NK-92 cells retain vitality and functionality when grown in standard cell culture conditionsPLOS ONE

Dear Dr. Kotzur,

Thank you for submitting your manuscript to PLOS ONE. After careful consideration, we feel that it has merit but does not fully meet PLOS ONE’s publication criteria as it currently stands. Therefore, we invite you to submit a revised version of the manuscript that addresses the points raised during the review process.

The manuscript was reviewed by two reviewers. One reviewer has recommended publication, but the other also suggest concerns in terms of strength of your manuscript. In addition, this associate editor has some suggestions. First, the introduction should be more concise. It looks redundant with descriptions on clinical trials that are not directly relevant to the purpose of the comparison study. Second, to minimize necessary supplement or treatment is important for cost effectiveness, however, data obtained through preclinical studies using GMP-compliant reagents will eventually required for the next step. One may not expect products prepared in petri dishes will be administered to patients even in early phase of study. Careful planning could be also critical.

Please submit your revised manuscript by Dec 03 2021 11:59PM. If you will need more time than this to complete your revisions, please reply to this message or contact the journal office at plosone@plos.org. When you're ready to submit your revision, log on to https://www.editorialmanager.com/pone/ and select the 'Submissions Needing Revision' folder to locate your manuscript file.

Please include the following items when submitting your revised manuscript:

- A rebuttal letter that responds to each point raised by the academic editor and reviewer(s). You should upload this letter as a separate file labeled 'Response to Reviewers'.
- A marked-up copy of your manuscript that highlights changes made to the original version. You should upload this as a separate file labeled 'Revised Manuscript with Track Changes'.
- An unmarked version of your revised paper without tracked changes. You should upload this as a separate file labeled 'Manuscript'.

If you would like to make changes to your financial disclosure, please include your updated statement in your cover letter. Guidelines for resubmitting your figure files are available below the reviewer comments at the end of this letter.

If applicable, we recommend that you deposit your laboratory protocols in protocols.io to enhance the reproducibility of your results. Protocols.io assigns your protocol its own identifier (DOI) so that it can be cited independently in the future. For instructions see: https://journals.plos.org/plosone/s/submission-guidelines#loc-laboratory-protocols. Additionally, PLOS ONE offers an option for publishing peer-reviewed Lab Protocol articles, which describe protocols hosted on protocols.io. Read more information on sharing protocols at https://plos.org/protocols?utm_medium=editorial-email&utm_source=authorletters&utm_campaign=protocols.

We look forward to receiving your revised manuscript.

Kind regards,

Yoshiki Akatsuka

Academic Editor

PLOS ONE

Journal Requirements:

When submitting your revision, we need you to address these additional requirements.

1. Please ensure that your manuscript meets PLOS ONE's style requirements, including those for file naming. The PLOS ONE style templates can be found at 

https://journals.plos.org/plosone/s/file?id=wjVg/PLOSOne_formatting_sample_main_body.pdf and 

https://journals.plos.org/plosone/s/file?id=ba62/PLOSOne_formatting_sample_title_authors_affiliations.pdf

2. Thank you for stating the following in the Acknowledgments Section of your manuscript: 

This work was supported by a network grant of the European Commision (H2020-MSC_ITN_765104_MATURE-NK) to the Mandelboim lab, the Israel Innovation Authority (Kamin grant), the Israel Science Foundation (Moked grant), the GIF Foundation, the ICRF professorship grant, the ISF Israel- China grant, the MOST-DKFZ grant, and the Ministry of Science and Technology grant.

Please note that funding information should not appear in the Acknowledgments section or other areas of your manuscript. We will only publish funding information present in the Funding Statement section of the online submission form. 

Please remove any funding-related text from the manuscript and let us know how you would like to update your Funding Statement. Currently, your Funding Statement reads as follows: 

R. K. was awarded the 

H2020-MSC_ITN_765104_MATURE-NK grant from the European Commision

http://www.mature-nk.eu.

Furthermore, the work was funded by

the Israel Innovation Authority Kamin grant 62615 http://innovation-israel-en.mag.calltext.co.il, 

the Israeli Science Foundation ISF Moked grant 442-18 https://www.isf.org.il/#/, 

the German-Israeli Foundation for Scientific Research and Development grant 1412-414.13/2017 http://www.gif.org.il/Pages/default.aspx, 

ICRF Professorship grant Israeli Cancer Research Fund https://www.icrfonline.org/grants/, 

the Israeli Science Foundation ISF China grant 2554/18 https://www.isf.org.il/#/,

the Ministry of Science and Technology Foundation - Deutsches Krebsforschungszentrum MOST-DKFZ grant 3-14931 https://www.trdf.co.il/eng/kolkoreinfo.php?id=8571, 

the Ministry of Science and Technology grant 3-14764 https://www.gov.il/he/Departments/ministry_of_science_and_technology.

The funders had no role in study design, data collection and analysis, decision to publish, or preparation of the manuscript.

Please include your amended statements within your cover letter; we will change the online submission form on your behalf. 

[Note: HTML markup is below. Please do not edit.]

Reviewers' comments:

Reviewer's Responses to Questions

**Comments to the Author**

1. Is the manuscript technically sound, and do the data support the conclusions?

The manuscript must describe a technically sound piece of scientific research with data that supports the conclusions. Experiments must have been conducted rigorously, with appropriate controls, replication, and sample sizes. The conclusions must be drawn appropriately based on the data presented. 

Reviewer #1: Yes

Reviewer #2: Yes

**********

2. Has the statistical analysis been performed appropriately and rigorously? 

Reviewer #1: Yes

Reviewer #2: Yes

**********

3. Have the authors made all data underlying the findings in their manuscript fully available?

The PLOS Data policy requires authors to make all data underlying the findings described in their manuscript fully available without restriction, with rare exception (please refer to the Data Availability Statement in the manuscript PDF file). The data should be provided as part of the manuscript or its supporting information, or deposited to a public repository. For example, in addition to summary statistics, the data points behind means, medians and variance measures should be available. If there are restrictions on publicly sharing data—e.g. participant privacy or use of data from a third party—those must be specified.

Reviewer #1: Yes

Reviewer #2: Yes

**********

4. Is the manuscript presented in an intelligible fashion and written in standard English?

PLOS ONE does not copyedit accepted manuscripts, so the language in submitted articles must be clear, correct, and unambiguous. Any typographical or grammatical errors should be corrected at revision, so please note any specific errors here.

Reviewer #1: Yes

Reviewer #2: Yes

**********

5. Review Comments to the Author

Please use the space provided to explain your answers to the questions above. You may also include additional comments for the author, including concerns about dual publication, research ethics, or publication ethics. (Please upload your review as an attachment if it exceeds 20,000 characters)

Reviewer #1: The authors showed that NK-92 cells could be expanded in RPMI medium with minimum supplements instead of MEM-alpha-based medium with various supplements, although serum and IL2 were still needed. This information is valuable for researchers using NK-92 cells. It should be clarified whether the potential of NK-92 cells are maintained after long-term cultivation in the RPMI-based media.

Reviewer #2: In this manuscript, the authors investigate the impact of the culture medium on the growth, vitality, and functionality of NK-92 cells. NK-92 are presented as highly promising and versatile cells used for off-the-shelf immunotherapy in current trials around the world. The authors describe their approach by comparing the cells grown in three different conditions: 1. The widely recommended growth in specialized NK-92 medium in a standing ventilated T25 flasks, 2. The commonly in cell culture used RPMI medium enriched with IL-2, in a standing flask as well, and 3. The commonly used RPMI with IL-2 in regular cell culture dishes. During the work for this manuscript, the authors compare the vitality of the cells via MTT and CFSE assays, the overall appearance by FACS staining of commonly with NK-92 associated and activating receptors on the surface of the cells, and the functionality via employment of activation assays, read out by IFN secretion, and killing assays, targeting various cancer cell lines.

Several studies (Fernández et al., 2021, Cancers; Chrobok et al., 2019, Cancers) already claim to have used the RPMI medium supplemented with IL-2 as growth and maintenance medium for NK-92, but so far, no proof has been presented that the substitution of medium would not alter the functionality of the cells. The present study focuses on showing these differences and the influence of the chosen medium on the appearance and abilities of the cells. The authors convincingly illuminate that the medium exchange does not significantly influence the functionality and growth of NK-92 cells. During this study is has been shown that the media can be used interchangeably without a loss of activation and vitality. The presented data supports the claim made by the authors and delivers the needed evidence to safely change the long-established NK-92 medium to an optional medium and raise the commonly used RPMI medium supplemented with IL-2 to a recommended culture medium for NK-92 cells. This manuscript only requires small revisions and is highly recommended for publishing.

General remarks

• The authors should present another form of activation used for the activation assay depicted in Fig. 3. A bacterial molecule like LPS could enhance the significance of the findings.

• As well, another inflammation associated cytokine apart from IFN should be added as an output marker for activation of NK-92 cells in Fig. 3, for example TNF-.

• The authors show in Fig. 2 a depiction of selected surface markers known to be expressed on NK-92 cell, but in the manuscript text many more markers are mentioned. Please add more marker staining to complete the picture of unfazed cells after the medium exchange

**********

6. PLOS authors have the option to publish the peer review history of their article (what does this mean?). If published, this will include your full peer review and any attached files.

If you choose “no”, your identity will remain anonymous but your review may still be made public.

**Do you want your identity to be public for this peer review?** For information about this choice, including consent withdrawal, please see our Privacy Policy.

Reviewer #1: No

Reviewer #2: No

[NOTE: If reviewer comments were submitted as an attachment file, they will be attached to this email and accessible via the submission site. Please log into your account, locate the manuscript record, and check for the action link "View Attachments". If this link does not appear, there are no attachment files.]

While revising your submission, please upload your figure files to the Preflight Analysis and Conversion Engine (PACE) digital diagnostic tool, https://pacev2.apexcovantage.com/. PACE helps ensure that figures meet PLOS requirements. To use PACE, you must first register as a user. Registration is free. Then, login and navigate to the UPLOAD tab, where you will find detailed instructions on how to use the tool. If you encounter any issues or have any questions when using PACE, please email PLOS at figures@plos.org. Please note that Supporting Information files do not need this step.

# Author comment: Author response to Decision Letter 0

**DOI:** 10.1371/journal.pone.0264897.r002

2 Dec 2021

The introduction should be more concise. It looks redundant with descriptions on clinical trials that are not directly relevant to the purpose of the comparison study.

 We have shortened the introduction in accordance with the reviewer’s suggestion.

 To minimize necessary supplement or treatment is important for cost effectiveness, however, data obtained through preclinical studies using GMP-compliant reagents will eventually required for the next step. One may not expect products prepared in petri dishes will be administered to patients even in early phase of study. Careful planning could be also critical.

 The reviewer is, of course, correct that GMP compliance is required for clinical administration. One of the main points of this paper, however, is to enable expansion of the use of NK-92. GMP facilities are not accessible to many groups (largely including our own) and are certainly not required in the exploratory research we wish to encourage. We have put forward the concept of easier handling, but ultimately the investigators in a clinical trial should, of course, carefully consider whatever methods they choose to employ.

 Please ensure that your manuscript meets PLOS ONE's style requirements, including those for file naming. T

 We have made the necessary changes.

 Thank you for stating the following in the Acknowledgments Section of your manuscript: 

 This work was supported by a network grant of the European Commision (H2020-MSC_ITN_765104_MATURE-NK) to the Mandelboim lab, the Israel Innovation Authority (Kamin grant), the Israel Science Foundation (Moked grant), the GIF Foundation, the ICRF professorship grant, the ISF Israel- China grant, the MOST-DKFZ grant, and the Ministry of Science and Technology grant.

 Please note that funding information should not appear in the Acknowledgments section or other areas of your manuscript. We will only publish funding information present in the Funding Statement section of the online submission form. 

Please remove any funding-related text from the manuscript and let us know how you would like to update your Funding Statement. 

 We have made the necessary changes, thank you for pointing this out!

 It should be clarified whether the potential of NK-92 cells are maintained after long-term cultivation in the RPMI-based media.

 All experiments were carried out on NK-92 cells which had been cultured in the stated conditions for at least six months. This is now indicated in the manuscript.

 The authors should present another form of activation used for the activation assay depicted in Fig. 3. A bacterial molecule like LPS could enhance the significance of the findings.

 A variety of cellular targets, interleukins (alone or in combination), and LPS are all featured in Figure 3, as requested by the reviewer. 

 As well, another inflammation associated cytokine apart from IFN should be added as an output marker for activation of NK-92 cells in Fig. 3, for example TNF-a.

 Results of TNFα secretion are now included in the manuscript summed up in Supplementary Figure 1.

 The authors show in Fig. 2 a depiction of selected surface markers known to be expressed on NK-92 cell, but in the manuscript text many more markers are mentioned. Please add more marker staining to complete the picture of unfazed cells after the medium exchange.

 Please see the manuscript for the full, updated list in Ffigure 2.

Attachment Submitted filename: Response to reviewers.docx Click here for additional data file.

# Peer review: Decision Letter 1

**DOI:** 10.1371/journal.pone.0264897.r003

**Authors:** Manish S. Patankar

21 Feb 2022

NK-92 cells retain vitality and functionality when grown in standard cell culture conditions

PONE-D-21-23659R1

Dear Dr. Kotzur,

We’re pleased to inform you that your manuscript has been judged scientifically suitable for publication and will be formally accepted for publication once it meets all outstanding technical requirements.

Within one week, you’ll receive an e-mail detailing the required amendments. When these have been addressed, you’ll receive a formal acceptance letter and your manuscript will be scheduled for publication.

An invoice for payment will follow shortly after the formal acceptance. To ensure an efficient process, please log into Editorial Manager at http://www.editorialmanager.com/pone/, click the 'Update My Information' link at the top of the page, and double check that your user information is up-to-date. If you have any billing related questions, please contact our Author Billing department directly at authorbilling@plos.org.

If your institution or institutions have a press office, please notify them about your upcoming paper to help maximize its impact. If they’ll be preparing press materials, please inform our press team as soon as possible -- no later than 48 hours after receiving the formal acceptance. Your manuscript will remain under strict press embargo until 2 pm Eastern Time on the date of publication. For more information, please contact onepress@plos.org.

Kind regards,

Manish S. Patankar, Ph.D.

Academic Editor

PLOS ONE

Additional Editor Comments (optional):

Reviewers' comments:

Reviewer's Responses to Questions

**Comments to the Author**

1. If the authors have adequately addressed your comments raised in a previous round of review and you feel that this manuscript is now acceptable for publication, you may indicate that here to bypass the “Comments to the Author” section, enter your conflict of interest statement in the “Confidential to Editor” section, and submit your "Accept" recommendation.

Reviewer #1: (No Response)

**********

2. Is the manuscript technically sound, and do the data support the conclusions?

The manuscript must describe a technically sound piece of scientific research with data that supports the conclusions. Experiments must have been conducted rigorously, with appropriate controls, replication, and sample sizes. The conclusions must be drawn appropriately based on the data presented. 

Reviewer #1: (No Response)

**********

3. Has the statistical analysis been performed appropriately and rigorously? 

Reviewer #1: (No Response)

**********

4. Have the authors made all data underlying the findings in their manuscript fully available?

The PLOS Data policy requires authors to make all data underlying the findings described in their manuscript fully available without restriction, with rare exception (please refer to the Data Availability Statement in the manuscript PDF file). The data should be provided as part of the manuscript or its supporting information, or deposited to a public repository. For example, in addition to summary statistics, the data points behind means, medians and variance measures should be available. If there are restrictions on publicly sharing data—e.g. participant privacy or use of data from a third party—those must be specified.

Reviewer #1: (No Response)

**********

5. Is the manuscript presented in an intelligible fashion and written in standard English?

PLOS ONE does not copyedit accepted manuscripts, so the language in submitted articles must be clear, correct, and unambiguous. Any typographical or grammatical errors should be corrected at revision, so please note any specific errors here.

Reviewer #1: (No Response)

**********

6. Review Comments to the Author

Please use the space provided to explain your answers to the questions above. You may also include additional comments for the author, including concerns about dual publication, research ethics, or publication ethics. (Please upload your review as an attachment if it exceeds 20,000 characters)

Reviewer #1: (No Response)

**********

7. PLOS authors have the option to publish the peer review history of their article (what does this mean?). If published, this will include your full peer review and any attached files.

If you choose “no”, your identity will remain anonymous but your review may still be made public.

**Do you want your identity to be public for this peer review?** For information about this choice, including consent withdrawal, please see our Privacy Policy.

Reviewer #1: No

# Editor report: Acceptance letter

**DOI:** 10.1371/journal.pone.0264897.r004

**Authors:** Manish S. Patankar

28 Feb 2022

PONE-D-21-23659R1 

NK-92 cells retain vitality and functionality when grown in standard cell culture conditions 

Dear Dr. Kotzur:

I'm pleased to inform you that your manuscript has been deemed suitable for publication in PLOS ONE. Congratulations! Your manuscript is now with our production department. 

If your institution or institutions have a press office, please let them know about your upcoming paper now to help maximize its impact. If they'll be preparing press materials, please inform our press team within the next 48 hours. Your manuscript will remain under strict press embargo until 2 pm Eastern Time on the date of publication. For more information please contact onepress@plos.org.

If we can help with anything else, please email us at plosone@plos.org. 

Thank you for submitting your work to PLOS ONE and supporting open access. 

Kind regards, 

PLOS ONE Editorial Office Staff

on behalf of

Dr. Manish S. Patankar 

Academic Editor

PLOS ONE
